# Healthcare professionals’ experiences of eHealth in palliative care for older people: challenges, compromises and the price of dignity

**DOI:** 10.1080/17482631.2024.2374733

**Published:** 2024-07-11

**Authors:** Rada Sandic Spaho, Lisbeth Uhrenfeldt, Theofanis Fotis, Jorunn Bjerkan, Ingjerd Gåre Kymre

**Affiliations:** a Faculty of Nursing and Health Sciences, Nord University, Norway; b Department of Orthopaedic Surgery, Lillebaelt Hospital, Kolding, Denmark; c Department of Regional Health Research, Southern Danish University, Odense, Denmark; d School of Sport & Health Sciences, Centre for Secure, Intelligent and Usable Systems, University of Brighton, Bodo, UK

**Keywords:** Palliative care, eHealth, electronic health records, healthcare professional, home care services, hospital municipal, health information interoperability, aged, patient discharge summary, dignity

## Abstract

**Purpose:**

To explore whether and how eHealth solutions support the dignity of healthcare professionals and patients in palliative care contexts.

**Method:**

This qualitative study used phenomenographic analysis involving four focus group interviews, with healthcare professionals who provide palliative care to older people.

**Results:**

Analysis revealed four categories of views on working with eHealth in hierarchical order: *Safeguarding the patient by documenting—eHealth is a* *grain of support, Treated as less worthy by authorities—double standards, Distrust in the eHealth solution—when the “solution” presents a* *danger;* and *Patient first—personal contact with patients endows more dignity than eHealth*. The ability to have up-to-date patient information was considered crucial when caring for vulnerable, dying patients. eHealth solutions were perceived as essential technological support, but also as unreliable, even dangerous, lacking patient information, with critical information potentially missing or overlooked. This caused distrust in eHealth, introduced unease at work, and challenged healthcare professionals’ identities, leading to embodied discomfort and feeling of a lack of dignity.

**Conclusion:**

The healthcare professionals perceived work with eHealth solutions as challenging their sense of dignity, and therefore affecting their ability to provide dignified care for the patients. However, healthcare professionals managed to provide dignified palliative care by focusing on patient first.

## Introduction

Dignity can be described in multiple ways, as can its absence (Galvin & Todres, 2015). Caring for a dying patient when they are vulnerable, enabling them to feel worthy despite their vulnerability, is a way of providing dignified care. Dignity encompasses both relationship and experience (Galvin & Todres, 2015). Galvin and Todres describe various caring ways that healthcare professionals (HCPs) can use to restore and maintain palliative patients’ dignity by recognizing and relieving suffering and offering possibilities for the appreciation of small things (Galvin & Todres, 2013). However, HCPs’ ability to provide good patient care can be influenced by the synergy between their work environments difficulties, professional relationships and sense of self-worth (Combrinck et al., 2022), which can affect HCPs’ sense of professional dignity.

Palliative care “improves the quality of life of patients and that of their families who are facing challenges associated with life-threatening illness, whether physical, psychological, social or spiritual” (WHO, 2020). Working in palliative care and with patients in the end-of-life stage requires not only medical but also psychological skill sets, such as empathy and the ability to discuss painful news with the patient, answer difficult questions, and gain the patient’s trust (Bessen et al., 2019; Fernando & Hughes, 2019). These skills lead to better outcomes in clinical treatment in terms of compassionate care (Bessen et al., 2019). Building a relationship with mutual trust between healthcare professionals and palliative care patients is crucial in providing dignified care (Jakobsen & Lind, 2023).

HCPs are facing challenges in delivering palliative care, including the introduction of new digital health technologies, organizational challenges, fragmented health services, staff shortages, and emotional fatigue (Bessen et al., 2019; Bønes et al., 2022a; Melby & Håland, 2021). There is a global shortage amounting to millions of healthcare professionals (Liu et al., 2017) and a growing and ageing population (United Nations, 2019), with the older population having comorbidities (Guidelines, Network AI, 2020; Siemens et al., 2020). In 2019, the global population of people aged 65 years and older was projected to double by 2050 (United Nations, 2019). According to the World Health Organisation (WHO, 2020), 56.8 million people in the world need palliative care, including 25.7 million people in their last year of life, but unfortunately, only 14% of them receive it (WHO, 2020). In the future, most palliative care patients will be older people with multiple chronic conditions (Etkind et al., 2017; Nicholson et al., 2022).

To address some of these challenges, the European Commission has called for the digitalization of healthcare systems in the European Union (European Commission, 2018). Digital health consists of digital technological solutions, such as the Internet of Things, telemedicine, telehealth, wearable devices, mHealth, and eHealth (Koszalinski et al., 2019; WHO, 2018, 2022). eHealth, as part of digital health, refers to technologies used in health fields for secure and cost-effective communication, information management, education, and research (WHO). eHealth comprehends different healthcare technological solutions, applications and services, including electronic health records (Frennert et al., 2023; Öberg et al., 2018). Therefore, this study uses terms eHealth technology, services and eHealth solutions interchangeably, and eHealth when referring to them all as a concept. Healthcare professionals access patients’ information through electronic health records, an eHealth solution that stores patients’ health data. These data should be accessible by multiple care providers simultaneously, regardless of their geographic location or level of care (Ovid Technologies Inc, 2023a). Digital health technologies are part of routine palliative care and are considered essential by HCPs (May et al., 2022). Digital healthcare can offer possibilities for improving patient-centred care, reducing costs and increasing the effectiveness of healthcare systems (European Commission, 2018; Sandic Spaho et al., 2023). However, national healthcare systems use non-interoperable digital and eHealth solutions, which can hinder access to patient information and introduce confusion and gaps in patient information (European Commission, 2018).

The health conditions and needs of older patients can change rapidly (Jakobsen & Lind, 2023; May et al., 2022), especially in palliative care, making them dependent on a range of healthcare services (Fernando & Hughes, 2019; Liebig & Piccini, 2017; Melby & Håland, 2021; Mertens et al., 2021), including both primary care and hospital-based care (Bønes et al., 2022a; Kumlin et al., 2022; Liebig & Piccini, 2017; Melby & Håland, 2021; Ovid Technologies Inc, 2023b; Ovid Technologies Inc, 2023). Therefore, cooperation between HCPs and the seamless sharing of patient information is of great importance in palliative care, as it can prevent hospital readmissions, avoid problems with symptom management, and enable holistic patient care (Eriksen et al., 2020; Liebig & Piccini, 2017; May et al., 2022). This can help HCPs with different professional backgrounds, working in various healthcare facilities, to work together as a palliative team (Fernando & Hughes, 2019; Roth & Canedo, 2019; Siouta et al., 2016).

Interoperability, i.e., the ability of different technological systems, such as eHealth solutions, to exchange information, can facilitate the seamless exchange of patient information, decrease the work involved in maintaining documentation and ensure better patient care (Lehne et al., 2019). In contrast, if HCPs, specifically members of the palliative team, use eHealth solutions that are not interoperable and do not share patient health data, this creates an obstacle in the patient information flow, challenging the continuity of care (Bønes et al., 2022a; European Commission, 2018; Liebig & Piccini, 2017; Mertens et al., 2021). When communication of patient data is delayed or fragmented, poor care coordination and risks to patient safety can result (Bønes et al., 2022a, 2022b; La Rocca & Hoholm, 2017; Mertens et al., 2021). Unfortunately, palliative patients may be discharged from the hospital without sufficient information about their treatment plans (Liebig & Piccini, 2017; Mertens et al., 2021), which affects not only their subsequent treatment but also the dignity of both the providers and the recipients of care.

Galvin and Todres describe the complexity of dignity:

”*As human beings, we appear to carry a coherent and deep sense of everything that is gathered for us, how things are for us, spatially, bodily, temporally, in mood. This ‘ self-gathering’ is core to our sense of identity and has deep implications for dignity.*” (Galvin & Todres, 2015, p413)

Dignity represents our inner human feeling of self-value, worth,and can be ruptured or disturbed if we perceive ourselves as not attaining the standards we have created for ourselves. Dignity is thus individual, delicate, and more than just autonomy and respect; it is neither subjective nor objective, but relational and experiential (Galvin & Todres, 2015).

It is vital to explore how HCPs deal with the challenges in using already existing eHealth solutions (Carlqvist et al., 2021), such as sharing patient information (Bønes et al., 2022a; European Commission, 2018; La Rocca & Hoholm, 2017), especially in palliative care (Liebig & Piccini, 2017; May et al., 2022; Mertens et al., 2021), and how these challenges affect HCPs’ dignity and their ability to provide dignified palliative care for older people. Knowledge on this topic is sparse. There is limited evidence-based literature discussing dignity within digital health and scant research on access to electronic medical records in the context of dignity for HCPs and patients (Bjerkan & Uhrenfeldt, 2021). Therefore, this study explores whether and how eHealth solutions support HCPs’ and patients’ dignity in palliative care contexts.

## Research question:

How do HCPs experience working with eHealth solutions, and how do challenges in using eHealth affect their dignity and their ability to deliver dignified palliative care to older people?

## Method

We adopted a qualitative approach to data collection, using focus group interviews (Creswell & Poth, 2018). Focus groups were used as the open discussion provided a way for the participants to express different perspectives (Morgan, 1996). Phenomenography was chosen for the data analysis as it is a qualitative approach that enables understanding different experiences of a given phenomenon, in this case, working with eHealth solutions (Holmström et al., 2000). It is noteworthy that phenomenography orients not towards subjects’ or individual conceptions but rather the collection of different realities formed about the phenomenon. An individual can have multiple realities and perspectives that are meaningful to them (Friberg et al., 2000; Marton, 1981; Sjöström & Dahlgren, 2002). In this study, we asked HCPs working in palliative care to contribute with their perceptions of their work with eHealth solutions.

### Participants and data collection

The first author conducted four focus group interviews with a total of 16 healthcare professionals who worked with palliative care patients in Norway: four physicians, ten nurses, one physiotherapist, and one occupational therapist. The physicians and three nurses worked in the municipality’s short-stay hospital; two oncology nurses worked in the municipality’s coordination office; and five nurses, the physiotherapist, and the occupational therapist, worked in home care settings. As such, these groups represented a range of professional backgrounds, knowledge bases and roles in palliative teams at the primary care level. They also represented various services in primary care. Municipalities organize primary care in Norway, while four regional health authorities organize hospital-based specialized care (Bønes et al., 2022a).

All the participants had hospital-based experience with the hospitals’ eHealth solutions and the information flow these provide, and were able to compare these with the eHealth solutions they have been using for years in primary care settings. The participants had an average of 19.9 years of healthcare experience. They had 7.7 years of palliative care experience in primary care settings. Each focus group included participants who worked in a single institution, which contributed to an open discussion. Of the 16 participants, two were men and 14 were women. The groups included participants at the same level in the institutional hierarchy to avoid power relations (Bjerkan & Uhrenfeldt, 2021; Friberg et al., 2000) and encourage them to be open about their perceptions. Two interview groups were composed entirely of nurses; one consisted of physicians, and one was a mixed group with nurses, a physiotherapist, and an occupational therapist.

The first and last authors developed a semi-structured interview guide. They modified it after the first focus group was conducted with the physician group based on the background data gathered in this session. These data ensured more specific questions in the interview guide. The focus group interviews were conducted by the first author, followed by another researcher (mentioned in the Acknowledgements), who took notes on the group`s interactions and dynamics. All focus group interviews were held between October 2021 and March 2022, i.e., during the COVID-19 pandemic, which delayed the recruitment process and finding a suitable time for conducting the interviews.

The interviews lasted approximately 90 minutes; three were held in the participants’ workspaces during working hours, while the fourth was organized in an informal space after working hours. An open discussion was encouraged, allowing everyone to participate and share as much as they wanted. These discussions provided rich data and covered topics beyond this article’s scope.

### Data analysis

Phenomenographic analysis was adopted as an approach to analysing qualitative data, based on its ability to provide deeper meanings and understandings of the participants’ experiences and conceptions of the phenomenon (Dahlberg, 2014; Holmström et al., 2000; Larsson et al., 2003; Marton, 1981). Transcribed conversations were read several times by the first and last authors to establish an understanding of the overall content, and then analysed according to Larsson and Holmström’s process (Larsson & Holmström, 2007). The responses which addressed how HCPs felt while working with eHealth in palliative care were extracted and searched for *“what”* and *“how”* content. That content was then searched for statements which expressed what participants focused on while working with eHealth solutions, how they described their way of working, and how they created meaning in working with eHealth solutions (Larsson & Holmström, 2007). Further analysis, following Larsson and Holmström, involved finding patterns in participants’ answers to questions about what the core of the HCPs’ work with eHealth solutions in palliative care is, when they feel successful, and when they feel hindered in their jobs in providing palliative care. Special attention was paid to non-dominant ways of understanding as those enriched the analysis. Later, the categories were developed, which describe different collective ways of understanding. Participants gave examples of their work with eHealth solutions while providing palliative care. In phenomenography, some participants’ experiences can be placed in more than one category, as one participant might experience working with eHealth in several ways (Larsson & Holmström, 2007). According to the procedure outlined by Larsson and Holmström, after the categories are developed, metaphors are assigned to the categories; these are presented in the results section together with representative quotes from the focus groups (Larsson & Holmström, 2007; Larsson et al., 2003). Special attention was given to conceptions of dignity and dignified palliative care in the results for further discussion (Galvin & Todres, 2013, 2015). The categories have a hierarchical and mutually exclusive order (Marton, 1981). This means that the participants with experiences in the higher category comprehend the understandings from the hierarchically lower category, but not vice versa. This hierarchical order forms the outcome space, in which the categories are hierarchically mutually related (Larsson & Holmström, 2007; Larsson et al., 2003).

To ensure the quality and trustworthiness of the research, the recommended steps were followed rigorously during the analysis and through discussions and reflections with all authors of this study (Larsson & Holmström, 2007; Larsson et al., 2003; Lincoln & Guba, 1985).

### Ethical considerations

This research has ethical approval from the Norwegian Centre for Research Data (NSD) with project number 438862. The research was then assessed by INNOVATEDIGNITY’s Ethical Scrutiny and Advisory Board project number 813928, which approved that ethical demands are in accordance with applicable legislation and international ethical guidelines. All participants were recruited through institutional gatekeepers and given an explanation of the research before signing the informed consent form. They were informed of their right to withdraw at any time. The conversations were recorded using the Dictaphone application and directly stored on a secure server at the University of Oslo (University in Oslo, 2023; University of Oslo, 2022). During transcription, the participants were anonymized. The coding key was stored separately from the recordings.

## Results

**Figure 1.**
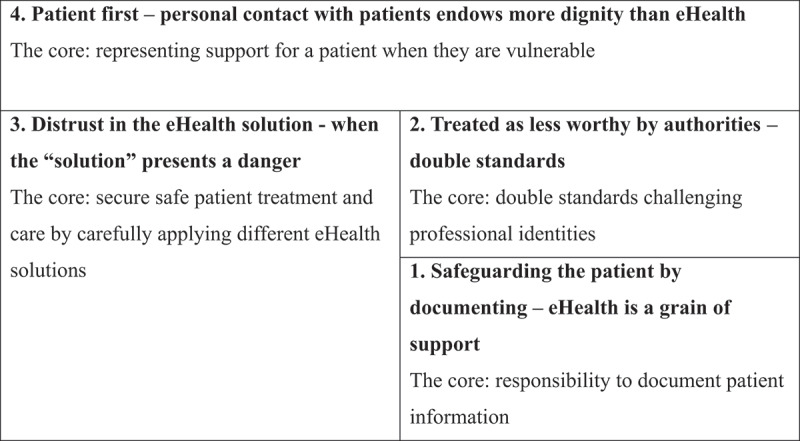
Hierarchical order of the HCPs’ conceptions of the work with eHealth.

The participants considered work in palliative care with older palliative patients to be uniquely sensitive based on patients’ vulnerability, limited lifespans, comorbidities, and rapidly changing health conditions and needs. Having up-to-date digital information and the ability to coordinate efficiently between the various health services working with the patient is crucial for palliative patients. The participants often expressed concerns that these needs were not met by the systems in place. The perspectives expressed by the participants are presented below in four categories in hierarchical order. This hierarchy means that HCPs whose conceptions are shown in the first category, *Safeguarding the patient by documenting—eHealth is a grain of support*, did not also express any of the perspectives shown in the other categories. On the other hand, the HCPs in the fourth category, *Patient first—personal contact with patients endows more dignity than eHealth*, expressed all four concepts, in varying intensities and ranges. The outcome space is presented in Figure 1, representing how the categories are related, similar to Larsson and Holmström’s presentation (Larsson & Holmström, 2007). The fourth category is highest in the hierarchy, and incorporates the other three. The second category incorporates the concepts described in the first, but not the third or fourth.

### The categories in hierarchical order

#### Safeguarding the patient by documenting—eHealth is a grain of support

The core of this category is the responsibility to document everything done, record information about the patient’s health status, safely monitor the patient, and share information with colleagues. Thus, the focus is on the patient’s state of health and what is needed to provide optimal care, safety, and symptom and pain management for the patient, supported by technology.

We are responsible for documenting everything we do, even if there is a failure in the system or if we don’t have access to the information. Collecting missing information from the hospital takes a lot of time, as we must call them and document our talk afterwards. It should be simple, but we must do it this way. We know there are limitations in access, but systems are safe, and healthcare professionals will only retrieve the information they need. We have a duty to maintain confidentiality. (Participant 8)

The participants consider eHealth a suitable approach to documenting the work and a safe tool for securing and sharing information. However, since eHealth solutions, and electronic health records, used by different levels of care are not interoperable, nobody in the healthcare system has up-to-date information. eHealth is fragmented, and in their daily work HCPs use many different eHealth solutions, such as: electronic health records, primary care eHealth solution, patient’s medication use (which is pharmacy shops’ eHealth solution), messaging with other HCPs, journal system that contains only some critical patient information and is available to all HCP, etc. Therefore, eHealth is conceptualized as an essential foundation for managing patient information, but not necessarily one that is sufficient on its own in all cases. The participants emphasized that they cannot rely on electronic health records, which should contain all patient health data, including illnesses and treatments across various health providers. Consequently, they must use other ways of collecting missing information, such as using other eHealth solutions, asking and talking with the patient, conducting clinical observation, and dialogue with colleagues. The oncology nurses knew their colleagues in the palliative team from other health services, so they knew who to call. However, home care nurses work with various patients, not just palliative ones. Hence, they had to call hospitals and other health services to find out where the patient was, as this information was not visible to them from the eHealth solutions they use. As such, the system challenges palliative team members’ personal sense of responsibility and expands their temporal workflow by creating new work demands.

#### Treated as less worthy by authorities—double standards

All the participants had previously worked in a hospital. They knew what hospital eHealth solutions could provide: a daily overview of each patient’s condition, the option to enter changes in the patient’s condition and measurements as they happen, access to diagnostics and measurements, and the chance to double-check important information. This gave them the sense of having a complete overview, safety at work, self-dignity, and self-efficacy.

However, the eHealth solution they currently work with does not provide any of these features. Once the report about patient’s health status is entered in the eHealth solution, it cannot be revised. The eHealth solution in use does not allow revision. If the patient’s health condition changes afterwards, the change can be entered only in some scattered fields, like notes, and therefore can be missed. Therefore, the nurses must memorize measurements or changes in patient status, medication doses, etc. and enter the report at the end of their workday. This causes delays in updates, which influences doctors’ actions and patient treatment. There is no possibility of double-checking or having an overview. Instead, because of the eHealth solution’s design, important information may not be visible. Reports must be forwarded to other health services, even those using the same solution, so reports become visible to them. The participants believed they needed to find solutions to these challenges on their own in order to work efficiently. The HCPs emphasized that they are not resistant to the technology, as they are aware of the possibilities it can provide.

The participating HCPs expressed a feeling that hospitals are in a privileged position, not just because of the “superior” eHealth systems they have access to, but because the patient discharge summary produced by the hospital directs the treatment plan that primary care should implement. However, as per governmental policy, digital communication is one-way, from the hospital to primary care, and not two-way. Also, there is no national standardization for interoperability and sharing data between primary care and hospitals’ eHealth solutions, not even when the eHealth solutions for primary care and for hospitals are made by the same company which makes it a national problem. Municipalities decide which eHealth solutions will be bought, a decision into which HCPs have no input. This places HCPs in a dependent position relative to the hospital, wherein they constantly have to call to obtain additional information because electronic communication is impossible. The HCPs considered eHealth solutions to be systems dividing “us” (primary care) and “them” (the hospital). The HCPs believed they were forced to work with the *“crippled”* eHealth solution, disempowered, hurt, neglected and treated as though their work and value were less than those of the hospital workers.

The HCPs’ perceptions of the double standards imposed on them varied. Some accepted the situation as *“that’s the way it has to be”* and *“We do what we can with what we have”*. In contrast, others were frustrated: *“Everything is coming late, measurements, reports”*, “*We have to constantly hustle the hospital to send the report”*, and expressed feelings of despair: *“We tried everything, but neither IT nor the municipality listens to our suggestions for improvements”*. IT is referred here to eHealth software designers. The participants felt that authorities’ double standards challenged their professional identities by treating some HCPs less worthy than others.

#### Distrust in the eHealth solution—when the “solution” presents a danger

At least half of the interviewed HCPs were concerned about whether they could trust the information in the eHealth solutions. The core aim these participants expressed was to secure safe patient treatment and care by thoroughly reviewing different eHealth solutions to gain a detailed overview of each patient’s health status, measurements and medications. However, the eHealth solution’s design makes it difficult to follow patients’ histories, and important information might be missed across the many scattered notes. Two groups were horrified that it is possible in the eHealth solution to read the information about one patient and, continued from this, the medication list of another patient without notifying the HCPs. They experienced this as the most frightening part of this eHealth solution that integrated electronic health records. One group emphasized that the written reports might fail to be uploaded to eHealth without professionals’ knowledge, or might be uploaded and later disappear from the system without a trace. These experiences raised serious questions about patient safety. To ensure patient safety, HCPs must always be aware of all relevant information, and they cannot trust an eHealth solution that may not provide this.

Another safety concern arose from the fact that palliative patients’ discharge summaries from the hospital are delayed by several days or a week. The palliative team in primary care is responsible for providing the care and treatment suggested by the hospital, but can struggle to do this without a simultaneous patient discharge summary.

Things are difficult for palliative patients because they often go to a hospital, and their treatment gets adjusted, and then here [in the municipality hospital], we make adjustments. We cannot communicate our adjustments to them [the hospital]. Then, when the patients go to the hospital again, they have the old [hospital’s] list. Their medication list is incorrect, and they make new adjustments. That`s not the same as I prescribed. So, it`s complete chaos for the patient, us, and the home care nurse who gives them this medicine. All because it is terribly communicated. (Participant 14)

Healthcare professionals often address the missed updates in communication by asking patients and their families to confirm information. They may also call the hospital or the patient’s GP or check the pharmacy’s eHealth solution, but do not feel that this is satisfactory. They are all aware that if a patient is discharged without a patient discharge summary, they cannot expect rapidly updated information, and have to rely on phone calls with the hospital.

When a new patient comes from the hospital and does not have a patient discharge summary with a medication list, we are scared and frustrated, and we lose lots of time finding the information that we need, and we have to call to get that information. We are also aware that we can get the wrong information over the phone, but the patient can’t wait. (Participant 9)

Delays in receiving information from the hospital hinder updated treatment and safe, timely and dignified care. The HCPs we interviewed overcame this not by use of eHealth but via phone calls, which caused an increased workload. The home care nurses “solved” these problems by phone, or if they knew they had a very sick patient and the eHealth solution was not functioning (for example, if they were unable to log in, or a report was missing), they visited the patient at home to check on them. Nurses from the municipality’s hospital have a daily routine of verbally reporting to each other and the physicians in the institution. The physicians in the municipality’s hospital used their professional network and all available eHealth solutions to get the feeling they obtained an overview. Distrust in the eHealth solution necessitated verbal reporting, which strengthened collaboration but increased the HCPs’ workload. All the interviewed HCPs had basic training from IT support. However, whenever HCPs asked for guidance for advanced functions in eHealth solution, they were directed back to the basic video instructions. HCPs found these instructions useless, as their level of knowledge of the work with that eHealth solution was higher than instructions offered by IT support. This caused HCPs to feel left out without any IT support. Especially as they previously had successful cooperation with the IT support for hospitals’ eHealth solution where they could even require personal changes in the interface of the eHealth solution. Distrust in the eHealth system affected participants’ professional sense of dignity. At the same time, the distress they felt at the workplace caused them to feel as if the workspace didn’t provide a a safe environment.

#### Patient first—personal contact with patients endows more dignity than eHealth

The core of palliative care is symptom management, especially pain relief. We do everything for end-of-life patients, so they have a good, dignified death, both for themselves and their families. To be with the patient is often more dignified than to check dosages of the medications. (Participant 6)

The participants felt that eHealth could enhance dignity with proper use and skills, but it undermined dignity if it led to lapses in patients’ pain relief, physical or mental suffering. HCPs emphasized that palliative care patients cannot wait several days for test results or messages from the hospital or GPs, as this may be too late for the information to make a difference. Therefore, having digital, up-to-date access to information on hospital diagnostics and patients’ responses to medications could avoid a repeat of painful procedures and provide dignity for the patient while saving time and costs. The participants also emphasized that having this information would spare palliative weak patients from having to repeat this information to each HCP individually.

Many HCPs, when talking about palliative patients, used the term “my patients” and mentioned that they have unique relationships with both patients and family members because “*we are with them till the end”*. They stated that there is nothing general in palliative care; palliative care is individualized care, adapted through deep, trustful relationships, non-verbal communication, and understanding each patient individually, without too many HCPs around the patient.

The most important is that the patient feels safe and taken care of when he can’t do it himself. So, we are his support, and dignity is of primary importance in palliative care. (Participant 3)

An example of providing dignified care is home care nurses arranging to spend a night at the dying patient’s home so that family members can rest during the night. The municipality’s hospital, for short stays, permits family members to visit the patient whenever they want and even to sleep in the patient’s room. Out of respect for the patient, HCPs do not document anything while they are with the patient, but do so afterwards. This is because they want to focus on being with the patient: The patient comes first, and reports come afterwards.

The participants described finding job satisfaction in placing the patient in focus, working as a team, and enabling dignified palliative care. However, the price they must pay is distress and time-consuming activities for collecting pieces of critical information. Distress is expressed in a range of ways, from discomfort to embodied sorrow, and does not end with the workday but is carried home, affecting the HCP’s whole life.

## Discussion

This study examined how HCPs experience their work with eHealth solutions and whether eHealth solutions support dignity in the palliative care context. The categories in the Results section present the concepts expressed in a hierarchical order, from initially seeing work with eHealth in terms of the responsibility to document one’s own work, to feeling disempowered by authorities’ double standards and facing the everyday challenges with eHealth solutions that frighten HCPs, to comprehensive feelings that the patient comes first and eHealth comes last. These results are ambiguous because of the challenges and compromises HCPs experience and because they raise the question of whether caring can be dignified despite these challenges. Galvin and Todres describe seven kinds of dignity: spatial, identity, interpersonal, temporal, mood, embodied and finitude dignity. These kinds of experiential and relational dignity have ranges and intensities and are interrelated and intertwined (Galvin & Todres, 2015). This approach describes indignity as a rupture of dignity and suggests ways of restoring human dignity (Galvin & Todres, 2015) and ensuring dignity in care settings (Galvin & Todres, 2013, 2015). Therefore, the findings from this study are discussed in the light of those kinds of dignity to untangle the experiential and relational challenges.

Temporal dignity encompasses the feeling of being competent, “I can”, and a sense of rightness in how things unwind towards the future (Galvin & Todres, 2015). The findings from this study show that HCPs experience an increased workload because of eHealth solutions and have to spend substantial amounts of time finding missing patient information. Even though it challenges their temporal dignity, their sense of responsibility made them feel that it was right to use that time as that increased patients’ safety in a holistic, caring way, preventing adverse effects. Temporality becomes a matter of dignity when vital information is delayed, especially for palliative patients, for whom up-to-date information is particularly important.

Having spatial dignity means that a person feels at home, supported by their place in the world. The environment supports the person and their identity, providing a feeling of worth and dignity (Galvin & Todres, 2015). The results show that HCPs provide spatial dignity for patients by spending the night at patients’ homes, allowing the families to sleep in patients’ hospital rooms, and visiting patients at home. On the other hand, when HCPs should have felt supported at work, this kind of support was lacking from the workspace, management, eHealth systems, and authorities. HCPs’ efforts were not recognized, leaving them to find solutions on their own. This caused a shift in their sense of mood dignity, where instead of peacefulness in their work, they experienced various levels of agitation and frustration, from losing time on documentation and searching for information to feeling hopeless, despairing, and fear, causing distrust in eHealth, the workspace, and the authorities, but also themselves. Their sense of identity dignity as healthcare professionals was ruptured, causing them to feel less worthy than colleagues from the hospital. According to Galvin and Todres, when an occupational group receives recognition for their contribution, that group’s identity dignity is enhanced (Galvin & Todres, 2015).

It is possible for the authorities to act to restore HCPs’ identity dignity. This calls for interpersonal dignity, defined as interactions which are respectful of others’ value and sensitive to their vulnerabilities (Galvin & Todres, 2015). The HCPs in this study valued patients’ vulnerabilities and their finitude of life by creating trustful and meaningful relationships, and recognizing and fulfilling patients’ needs without much verbal communication. However, the need to constantly call hospitals to obtain missing information made HCPs more vulnerable as they had no support, relied on their own networks, and felt exposed.

When a person feels an embodied sense of capability, “I can and am”, and simultaneously accepts vulnerability as dignified, we can say they possess embodied dignity (Galvin & Todres, 2015). By placing the patient in focus and recognizing the value of their limited time, HCPs provide support and dignified palliative care for older patients. On the other hand, HCPs experience that the work in palliative care takes a toll in terms of bodily discomfort and the embodied sorrow that they carry with them even when not at work. This cost of HCPs’ own embodied dignity might be left unrecognized by others.

Galvin and Todres propose ways of restoring dignity, such as public and social reintegration and rebuilding of justice (Galvin & Todres, 2015). This means that supporting HCPs and reducing the challenges they experience on a daily basis can restore their self-value and trust in the healthcare system. Despite all the challenges that disturb HCPs’ sense of own dignity and their own feelings of powerlessness, they do everything in their power within the constraints they experience in their work to provide dignified palliative care. Without their devotion to correctly doing their job, palliative care could not be dignified.

Various studies have demonstrated similar challenges with different non-interoperable eHealth solutions that increase HCPs’ workload and are invisible to employers, such as a lack of organizational support and delayed discharge summaries, which cause frustration, fragmented care and jeopardized patient safety, reduced job satisfaction for HCPs, and a lack of sufficient time with patients (Andersen, 2019; Bjerkan et al., 2021; Fernando & Hughes, 2019; Frennert et al., 2023; Helse-og omsorgsdepartementet Ministry of Health and Welfare, 2019; La Rocca & Hoholm, 2017; Mertens et al., 2021; Öberg et al., 2018). Adding more eHealth solutions is not a solution, as it can lead to HCPs facing digital chaos (Frennert et al., 2023; Öberg et al., 2018) together with feelings of being overstretched and conflicted between caring touch and task performances and guarding patient safety, and can lead many nurses to leave the profession (Brall et al., 2019; Combrinck et al., 2022; Frennert et al., 2023 ; Liu et al., 2017; Nav, 2023). On the other hand, when patient information is up-to-date and available to all HCPs, this ensures patient safety, prevents medical errors, avoids the unnecessary repeating of examinations, and lessens the documentation burden while improving patient symptom management and communication between HCPs and patients (Lehne et al., 2019; May et al., 2022).

Our study offers a view of these challenges from the perspective of an HCP’s experiential and relational dignity. The dignity perspective provides an understanding of the ambiguous lived experiences of eHealth in palliative care for older people and, in addition, shows how various kinds of dignity are intertwined in this understanding.

## Strengths and limitations

It might be viewed as a limitation that this study has a small number of participants, making it hard to generalize the findings. However, the participants do represent a diverse range of perspectives: participants were from both urban and rural areas, worked in different healthcare services and had diverse professional backgrounds. Furthermore, all the interviews were conducted in person, which enabled detailed field observations.

It is a strength that the participants in this study had experience with two of the three possible eHealth solutions for primary care in Norway. The experiences with both eHealth solutions were similar, and non-interoperability is a nationwide and even worldwide problem. Similar findings from other European countries underpin the importance of our findings as a much broader problem that needs to be untangled.

This study contributes to raising awareness of the importance of recognizing HCPs’ dignity in providing care, which has thus far been overlooked and taken for granted.

## Conclusion

The current status with eHealth cannot support dignified palliative care. The work with eHealth systems was a starting point for collecting pieces of information that must be supplemented through additional data collection and communication to provide HCPs with all necessary information and ensure patient safety. Everyday eHealth challenges jeopardize HCPs’ dignity and their sense of professional worth by causing them to feel less valuable, making them embody sorrow, exposing their vulnerabilities and leaving them to find solutions on their own. However, despite these challenges, HCPs safeguard patients’ dignity and provide dignified palliative care for older people.

## Future implications

The findings from this study could guide possible future actions for providing dignified palliative care in connection with eHealth solutions. Making eHealth solutions interoperable and reliable, with access to up-to-date information, and acknowledging healthcare professionals’ work ethic and duty of confidentiality could make everyday work more dignified for healthcare professionals. This, in turn, would restore their trust in the healthcare system, make it easier to follow the patient through different levels of care, create trust and better relations between different care levels, safeguard patient treatment and care, and give HCPs more time to be present with the patient, hearing and fulfilling their needs at the right time when the patient needs it, consequently ensuring holistic, dignified palliative care. The on-site IT support to the HCPs and the eHealth system could be helpful. Therefore, it is necessary to involve all stakeholders, for example, through systematizing law requirements for eHealth solutions, promoting cooperation between the IT sector and the solutions’ users, both healthcare professionals and patients. Specifically, collaboration between IT companies will enable interoperability between different eHealth solutions, thereby ensuring more user-friendly, useful and practical digital solutions for palliative healthcare.

## Data Availability

This article is an Open Access article and can be used, distributed, or adapted under terms of the Creative Commons Attribution 4.0 International License (http://creativecommons.org/licenses/by/4.0/). You must provide credit to authors and sources, provide a link to the Creative Commons license, and indicate if changes were made.

## Abbreviations

HCPs: healthcare professionals
WHO: World Health Organisation
IT: software designer professionals, in this study connected to designing eHealth solutions

## Consortia

On behalf of INNOVATEDIGNITY, http://www.innovatedignity.eu
